# Exploring the importance of sulfate transporters and ATP sulphurylases for selenium hyperaccumulation—a comparison of *Stanleya pinnata* and *Brassica juncea (Brassicaceae)*

**DOI:** 10.3389/fpls.2015.00002

**Published:** 2015-01-23

**Authors:** Michela Schiavon, Marinus Pilon, Mario Malagoli, Elizabeth A. H. Pilon-Smits

**Affiliations:** ^1^Department of Agronomy, Food, Natural Resources, Animals and the Environment, University of PadovaLegnaro, Padova, Italy; ^2^Department of Biology, Colorado State UniversityFort Collins, CO, USA

**Keywords:** *Brassica juncea*, *Stanleya pinnata*, selenium, sulfur, uptake, ATP-sulphurylase, gene expression

## Abstract

Selenium (Se) hyperaccumulation, the capacity of some species to concentrate Se to levels upwards of 0.1% of dry weight, is an intriguing phenomenon that is only partially understood. Questions that remain to be answered are: do hyperaccumulators have one or more Se-specific transporters? How are these regulated by Se and sulfur (S)? In this study, hyperaccumulator *Stanleya pinnata* was compared with related non-hyperaccumulator *Brassica juncea* with respect to S-dependent selenate uptake and translocation, as well as for the expression levels of three sulfate/selenate transporters (*Sultr*) and three ATP sulphurylases (*APS*). Selenium accumulation went down ~10-fold with increasing sulfate supply in *B. juncea*, while *S. pinnata* only had a 2–3-fold difference in Se uptake between the highest (5 mM) and lowest sulfate (0 mM) treatments. The Se/S ratio was generally higher in the hyperaccumulator than the non-hyperaccumulator, and while tissue Se/S ratio in *B. juncea* largely reflected the ratio in the growth medium, *S. pinnata* enriched itself up to 5-fold with Se relative to S. The transcript levels of *Sultr1;2* and *2;1* and *APS1, 2, and 4* were generally much higher in *S. pinnata* than *B. juncea*, and the species showed differential transcript responses to S and Se supply. These results indicate that *S. pinnata* has at least one transporter with significant selenate specificity over sulfate. Also, the hyperaccumulator has elevated expression levels of several sulfate/selenate transporters and APS enzymes, which likely contribute to the Se hyperaccumulation and hypertolerance phenotype.

## Introduction

Selenium (Se) is an essential trace element for most animals and humans, who use selenocysteine as a component of at least 25 different selenoproteins, including a number of thioredoxin reductases and glutathione peroxidases (Rayman, 2009). Excess consumption of Se can be deleterious, however, because non-specific replacement of cysteine by selenocysteine in proteins disrupts protein function (Stadtman, 2000, 2005). The window between the amount of Se required as a nutrient (50–70 μg Se day^−1^, USDA, 2012) and the amount that is toxic is narrow; therefore, both Se deficiency and toxicity pose problems worldwide (Reilly, 2006). Selenium deficiency occurs where Se concentration in food crops is very low (Broadley et al., 2006) and may cause heart diseases, reduced fertility, hypothyroidism, and poor immune system function (Rayman, 2012); on the other hand, Se at high doses is toxic as it induces adverse cardiometabolic effects, as associated with an increased risk of type-2 diabetes and hyperlipidemia (Lee and Jeong, 2012; Rayman, 2012).

Plants may help to alleviate both Se deficiency and toxicity. They represent the principal source of dietary Se for a large part of the world population and can also be employed to remove excess Se from soils or wastewaters (phytoremediation) (de Souza et al., 1998; Van Huysen et al., 2003). Selenium has not been recognized to play an essential function in higher plants, although a number of beneficial effects via enhanced growth and antioxidant activity have been documented (Pilon-Smits et al., 2009; Saidi et al., 2014). Plants take up Se mainly in the form of selenate (SeO^2−^_4_) or selenite (SeO^2−^_3_). Selenate is the most abundant bioavailable form of Se in alkaline and well-oxidized soils and can be transported across plasma membranes through the activity of sulfate permeases owing to its chemical similarity to sulfate (Ellis and Salt, 2003; Sors et al., 2005). The selectivity of plant transport toward selenate and sulfate varies between plant species and is strongly associated with the sulfur (S) nutritional status of the plant (White et al., 2004). It has been proposed that at higher external sulfate availability, the selectivity of the constitutively expressed (low-affinity) plant transport system for sulfate over selenate is lower, while the high-affinity sulfate transporter system that is induced at low external sulfate availability has a higher selectivity for sulfate over selenate (White et al., 2004). Thus, different sulfate transporters in a single plant might exhibit different selectivity for sulfate vs. selenate.

The existence of a common mechanism for the uptake of selenate and sulfate in plants was first demonstrated in *Arabidopsis thaliana* mutants lacking a functional high-affinity sulfate transporter SULTR1;2. The mutation conferred to these plants significantly enhanced resistance to selenate (Shibagaki et al., 2002). SULTR1;2 has been proposed as the major transporter for influx of selenate into the plant root. At this point, it is still unclear whether AtSULTR1;2 has higher selectivity for selenate over sulfate. An additional high-affinity root sulfate transporter with much lower expression level is SULTR1;1 (Barberon et al., 2008).

Once entered into the plant cells, selenate is transported via the xylem to the leaf, which involves the low-affinity sulfate transporter, SULTR2;1 in the root and leaf vascular tissues (Hawkesford, 2003). There, selenate enters the sulfur reductive assimilation pathway. Like sulfate, selenate is believed to be activated by the enzyme ATP sulphurylase (APS), forming adenosine 5′-phosphoselenate (APSe). The APS gene family in *A. thaliana* has four members: APS1 (Leustek et al., 1994), APS2, APS3 (Murillo and Leustek, 1995), and APS4 (Hatzfeld et al., 2000). All isoforms are plastidic, but APS2 may also localize to the cytosol. APS1, 3, and 4 are subject to miRNA-mediated post-transcriptional regulation (Kawashima et al., 2009; Liang and Yu, 2010). Overexpression of APS1 in *Brassica juncea* has proven that the activation of selenate to APSe is one of the rate-limiting steps for selenate assimilation in plants (Pilon-Smits et al., 1999). Selenate is further reduced to selenite and assimilated into the selenoamino acids selenocysteine (SeCys) and selenomethionine (SeMet). The non-specific incorporation of these selenoamino acids into proteins, particularly replacing Cys by SeCys, is thought to cause disruption of their molecular structure and loss of their folding, leading to toxicity (Terry et al., 2000; Van Hoewyk, 2013).

Most plant species contain less than 25 μg Se g^−1^ dry weight in their natural environment and cannot tolerate much higher Se concentrations (White et al., 2004). These plants are called non-accumulators. In contrast, some species of the genera *Stanleya* (*Brassicaceae*) and *Astragalus* (*Fabaceae*) are classified as Se hyperaccumulators due to their capacity to accumulate over 1000 μg Se g^−1^ dry weight in their shoots (0.1–1.5%) while thriving on seleniferous soils containing only 2–10 ppm Se (Terry et al., 2000; Galeas et al., 2007; Pilon-Smits and LeDuc, 2009). A third category of plants, known as secondary Se accumulators, grow on soils of low-to-medium Se content and accumulate up to 1000 μg Se kg^−1^ dry (Terry et al., 2000). Examples of secondary accumulators are *Brassica juncea* and *Brassica napus*.

Selenium hyperaccumulators are also hypertolerant to Se. They have evolved several mechanisms to achieve tolerance to excess of this element. Firstly, methylation of SeCys can form the non-protein amino acid methyl-SeCys (MetSeCys), which is not incorporated in proteins (Neuhierl and Böck, 2002). Methylation of SeCys occurs also in non-accumulators, but to a very low extent. Met-SeCys can be further metabolized to volatile dimethyldiselenide in hyperaccumulators (Terry et al., 2000). Finally, Se hyperaccumulators show tissue-specific sequestration of Se in epidermal vacuoles, which may be a tolerance mechanism (Freeman et al., 2006, 2010). A recent study conducted in *S. pinnata* investigated the molecular mechanisms at the basis of Se tolerance and hyperaccumulation in this plant species (Freeman et al., 2010). Compared to the related non-hyperaccumulator *Stanleya albescens, S. pinnata* contained higher levels of antioxidants, of defense-related phytohormones, of selenocysteine methyltransferase and Met-SeCys, and revealed general up-regulation of sulfur assimilation.

While studies so far have given some insight into Se hyperaccumulation mechanisms, to date it is not known how Se hyperaccumulators are able to specifically take up and translocate Se over S. Hyperaccumulators are characterized by an elevated Se:S ratio, compared to other species and to their growth medium (White et al., 2004, 2007; Harris et al., 2014). Thus, in contrast to non-hyperaccumulators, hyperaccumulators appear to discriminate between sulfate and selenate for uptake, and preferentially accumulate Se over S. Additionally, Se hyperaccumulators showed a marked and S-independent seasonal variation in Se concentration in different plant organs, indicative of Se flow from roots to young leaves in early spring, from older to younger leaves and reproductive tissues in summer and from shoot to root in the fall (Galeas et al., 2007). Therefore, Se fluxes at the whole-plant level appear to be specialized in hyperaccumulators and distinct from S movement. To explain these phenomena, hyperaccumulator plants have been hypothesized to have altered regulation of sulfate/selenate transporters, and one or more transporters with enhanced selenate specificity (White et al., 2007; Harris et al., 2014).

As these physiological differences may be partly explained by differences in the selectivity for selenate and sulfate by the transporters involved, comparative studies on the sulfate transporters of hyperaccumulators and closely related non-hyperaccumulator species may provide useful insights into Se/S discrimination mechanisms. A better knowledge of these mechanisms at the molecular level are not only intrinsically interesting but might also help in the development of plants capable of sulfate-independent Se accumulation, through genetic engineering approaches. Such plants would be applicable in Se phytoremediation, which is often hampered by high sulfate levels.

Specific questions addressed in this study were: may Se-specific transporters exist in the hyperaccumulator *S. pinnata*? How are these regulated by the relative availabilities of selenate and sulfate in the growth medium? The main aim of this study was to dissect the roles of specific sulfate transporters in Se accumulation and sulfate/selenate discrimination in the hyperaccumulator *S. pinnata*, in comparison with the related non-hyperaccumulator *B. juncea*. The expression of *APS* genes was also investigated, since APS is a key enzyme for sulfate/selenate assimilation, and *Sultr* and *APS* genes are in some cases co-regulated via miRNA395 (Liang and Yu, 2010). Specific transcripts for sulfate transporters and ATP sulphurylase isoforms of both species were distinguished, and their expression was analyzed in relation to varying S and Se supply.

## Materials and methods

### Plant material and experimental design

Seeds of *B. juncea* and *S. pinnata* were surface-sterilized by rinsing in 70% (v/v) ethanol for 30–60 s, then in 5% (v/v) sodium hypochlorite (NaClO) for 30 min on a rocking platform, and finally washed in distilled water for 5 × 10 min. *Stanleya pinnata* seeds were obtained from Western Native Seed (Coaldale, Colorado). *Brassica juncea* was originally obtained from the US Department of Agriculture plant introduction station, as described before (Pilon-Smits et al., 1999).

The seeds were allowed to germinate on washed 2:1 Turface®/sand mixture in a grow room under fluorescent lights with a 16/8-h light/dark photoperiod. Seven day-old *B. juncea* and 3 week-old *S. pinnata* seedlings (same developmental stage) were transferred to 0.5 L-hydroponic containers, with a density of five plants per container. They received a complete half-strength Hoagland nutrient solution (Hoagland and Arnon, 1938), which contains 0.5 mM MgSO_4_.

After 7 days of growth under the conditions described above, plants of both species were grown in the same containers for 5 days under S-deficiency (same nutrient composition but without sulfate) to induce the high affinity sulfate transport system. Plants were then cultivated for 3 days in the presence of 0, 0.5, or 5 mM S, in combination with 0, 10, or 20 μM Se (added in the form of sodium selenate).

At the end of the experiment, the plants were harvested, immersed for 1 min in ice-cold distilled water to desorb sulfate/selenate that was attached to the root apoplast, and dried with blotting paper. Root and shoot samples (100–200 mg) from each plant were immediately frozen with liquid nitrogen and kept at −80°C for gene expression analyses, while the remainder of the plant was placed in a drying oven for 2 days at 50° for elemental analysis.

The experimental design for plant growth was randomized and for each experimental condition three replicates were performed.

### Determination of total Se and S in plants

Foliar and root tissues of *B. juncea* and *S. pinnata* plants were dried for 48 h at 50°C and then digested in nitric acid as described by Zarcinas et al. (1987). Inductively coupled plasma atomic emission spectroscopy (ICP-AES) was used as described by Fassel (1978) to determine each digest's elemental concentrations (Se, S).

For each experimental treatment, data obtained were the means of five measurements, each corresponding to one biological replicate. Data were expressed as mg element kg^−1^ dry weight.

### Expression analysis of genes involved in sulfate/selenate transport and assimilation

Quantitative real-time reverse transcription polymerase chain reaction (qRT-PCR) experiments were carried out to evaluate the expression of six genes involved in S/Se transport and assimilation. RNA was extracted from roots and leaves of *B. juncea* and *S. pinnata* plants of the following experimental conditions: S 0 Se 0, S 0 Se 20 μM, S 0.5 mM Se 0, S 0.5 mM Se 20 μM, S 5 mM Se 0, S 5 mM Se 20 μM. Each biological replicate was represented by a separate plant. RNA extraction was performed using a phenol/chloroform protocol according to Sambrook and Russell (2001). After DNAse treatment, cDNA was prepared from 3 μg of RNA per sample, using 200 U of Superscript Reverse Transcriptase III (Life Technologies) and oligodT as primer in 20 μl reaction volume. Mixtures were incubated at 37°C for 60 min, 70°C for 5 min, and 4°C for 5 min to stop the RT reaction. Specific primer pairs for each of the genes of interest as well as the actin 1 reference gene were designed on conserved sequences of *B. juncea* and other *Brassicaceae* spp. (Table 1) and tested for their activity at 58–67°C by conventional PCR. Quantitative Real-Time RT-PCR analyses were then performed using a thermal cycler (Roche 480) equipped with a 96 well plate system with the SYBR green PCR Master Mix reagent (Applied Biosystems). Each qPCR reaction was performed in a final volume of 10 μl containing 1 μl of cDNA diluted 1:10, 1 μL of each primer (10 mM), and 5 μl of 2× SYBR Green PCR Master Mix, according to the manufacturer's instructions. The following thermal cycling profile was used for all PCRs: 95°C for 10 min, 50 cycles of 95°C for 15 s, 60°C for 1 min. The analysis of expression of each biological replicate for each gene was evaluated in two technical replicates.

**Table 1:** **Sequences of primers used in qRT-PCR reactions**.

**Gene name**	**Forward primer 5′–3′**	**Reverse primer 5′–3′**
*Bj/SpSultr1;1*	TGTTCATCACACCGCTCTTC	TGCTGCGTCAATGTCAATAAG
*BjSultr1;2*	ATGGCTGGATGTCAAACTGC	TCAGAGGAATCACTGCGTTG
*SpSultr1;2*	TAGTGATTGCTGCGAGGATG	CGTCGTTCTCTTGACATTGC
*BjSultr2;1*	TTGGGCTACAAGAAACTCGTC	CTGAAAATCCCGAAAGAAGC
*SpSultr2;1*	CATCGCCGTCTCACACCC	ATCGTTGCCGTTGTTGCTTT
*Bj/SpAPS1*	CCCTATCCTTTTGCTTCATCC	GTGCTGCTTCATCCTCCAAC
*BjAPS2*	CATCAAGAGGAACATCATCAGC	TTACAGGCTATCTCCTAAACAGC
*SpAPS2*	CATCAAGAGGAACATCATCAGC	TTACAGGCTATCTCCAAAACAGC
*Bj/SpAPS4*	GAGAAGGTGCTTGAGGATGG	TTGGAGATGGGAAGATGGAG
*Bj/SpActin1*	AGCATGAAGATCAAGGTGGTG	CTGACTCATCGTACTCTCCCT

Quantitative RT-PCR analyses were performed on three biological replicates. All quantifications were normalized to the actin housekeeping gene and amplified in the same conditions. The obtained CT values were analyzed with the Q-gene software by averaging three independently calculated normalized expression values for each sample. Expression values are given as the mean of the normalized expression values of the biological triplicates, calculated according to Equation 2 of the Q-gene software (Muller et al., 2002).

### Statistical analysis

The software program JMP-IN (SAS Institute, Cary, NC) was employed for statistical analysis of metal tolerance and accumulation data. The data were checked for normal distribution and equal variance. ANOVA was performed followed by pairwise *post-hoc* analyses to determine which of the means differed significantly (α = 0.05). Statistically significant differences are reported in the text and shown in the figures.

## Results

### Effects of different Se/S ratios on Se and S accumulation in *B. juncea* and *S. pinnata*

**Figure 1:**
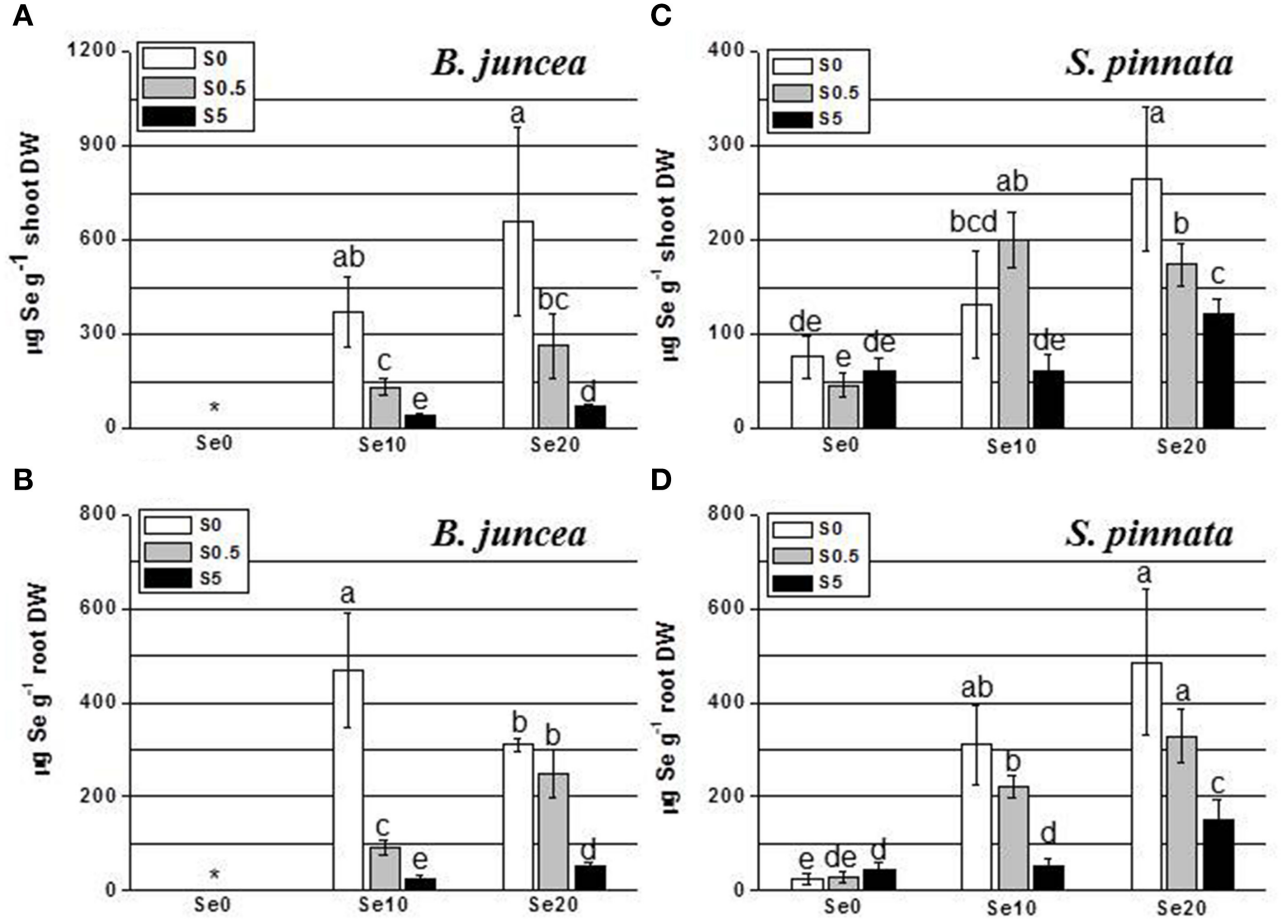
**Concentration of selenium (Se) in *B. juncea* and *S. pinnata* shoots (A,C, respectively) and roots (B,D, respectively) when plants were cultivated in the presence of different Se/S ratios**. All plants were pretreated for 5 days in nutrient solution without sulfate and then supplied for 3 days with 0, 10 or 20 μM selenate and 0, 0.5, or 5 mM sulfate. Data shown are the mean of five replicates ± SD. Letters above bars indicate significant differences between the means (*P* < 0.05). The asterisk indicate no significant differences among plants grown with different levels of sulfur in minus Se, and is referred to values significantly lower than those measured in Se-treated plants.

**Figure 2:**
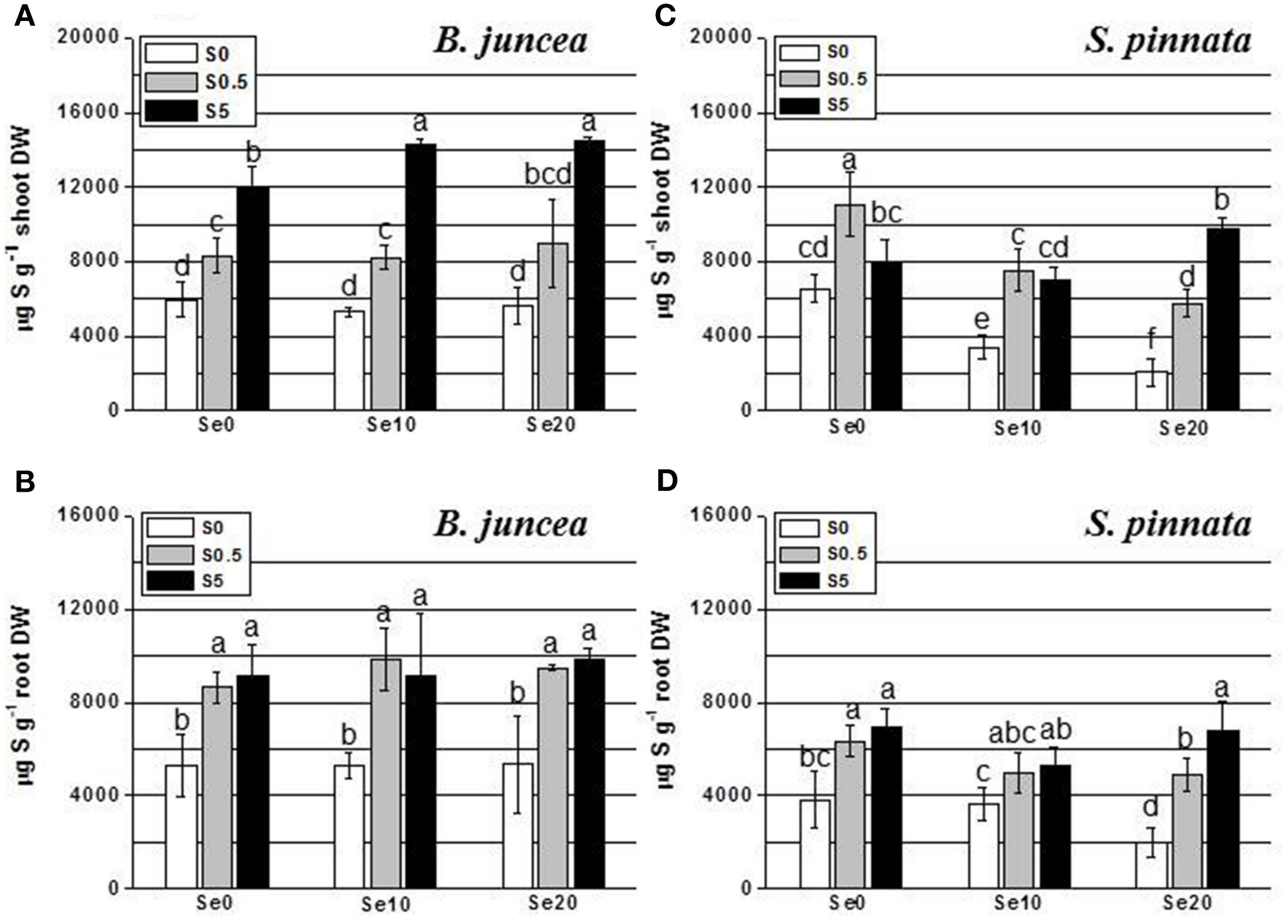
**Concentration of sulfur (S) in *B. juncea* and *S. pinnata* shoots (A,C, respectively) and roots (B,D, respectively) of plants pretreated for 5 days in nutrient solution without sulfate and then supplied for 3 days with 0, 10, or 20 μM selenate and 0, 0.5, or 5 mM sulfate**. Data shown are the mean of five replicates ± SD. Letters above bars indicate significant differences between the means (*P* < 0.05).

After 5 days of S starvation, hyperaccumulator *S. pinnata* and non-hyperaccumulator *B. juncea* were supplied with different concentrations of selenate (0, 10, 20 μM) and sulfate (0, 0.5, 5 mM), after which root and shoot Se and S accumulation were determined. The two species showed a differential pattern of Se and S accumulation in tissues depending on the Se/S ratios of the nutrient solution (Figures 1, 2). Two-Way ANOVA of Se accumulation in *B. juncea* and *S. pinnata* in relation to the S and Se dose applied revealed a significant species effect (Factor A), a significant effect of S and Se dose (Factor B), as well as a significant interaction effect (*P* < 0.05) for both the shoots (Tables 1S, 2S) and the roots (Tables 3S, 4S).

In the shoot of *B. juncea* plants, the trend of Se accumulation as a function of Se supply was linear under both S-deficiency and S-sufficient condition (Figure 1A). *B. juncea* plants accumulated more Se in the shoot when S was absent in the growth medium (from about 2.5- to 12-fold compared to S-sufficient plants). Generally, plants provided with high S (5 mM) accumulated the lowest amounts of Se in the shoot. In *B. juncea* roots (Figure 1B), Se accumulated linearly with Se supply when plants were supplied with 0.5 or 5 mM S but in S-starved plants, Se accumulation in the roots was maximal at an external Se of 10 μM (Figure 1B). Root Se levels showed a general inverse relationship with S supply. When elevated S levels (5 mM) were supplied to plants, very low values of Se concentration were measured in roots. This difference was maximal at a supply of 10 μM Se, where the root Se concentration of S-deplete plants was 8-fold higher than in plants supplied with 5 mM S.

In *S. pinnata* shoots, Se accumulation followed a different trend in response to the variation of the Se/S ratio in the nutrient solution, compared to *B. juncea* (Figure 1C). Appreciable amounts of Se were detected in *S. pinnata* shoot even when plants were not exposed to Se, and values were comparable between S-starved and S-sufficient plants. This Se must have been present in the seeds, which were wild-collected from seleniferous areas, and are indeed known to contain high Se levels in the field (Freeman et al., 2012). Although Se accumulation in *S. pinnata* shoots showed a slight trend to be inversely correlated with S supply, the negative effect of increasing sulfate levels on Se accumulation was only 2–3-fold, much less pronounced than for *B. juncea* (Figures 1A,C). *S. pinnata* generally attained lower Se levels than *B. juncea*, except when the plants were treated with excess S.

In *S. pinnata* roots, generally similar results were obtained as for the shoots (Figure 1D), except that a linear pattern of Se accumulation was observed also in plants that were S-deficient. There was a general trend for Se accumulation to reduce with external S level, but to a lesser extent than that seen in *B. juncea* roots, and in fact not significantly different between S-deplete and S replete (Figure 1D). Root Se levels were higher for *S. pinnata* than *B. juncea* for most treatments).

The level of S in the shoot of *B. juncea* was found to increase in response to increased sulfate concentration in the nutrient solution (Figure 2A). Interestingly, under conditions of excess S supply, Se treatment resulted in significantly higher shoot S levels. In the roots of *B. juncea* (Figure 2B), S accumulation did not vary with Se supply. The S-starved plants contained lower S levels than S-replete plants, as expected, without appreciable differences among plants supplied with different S concentrations (0.5 or 5 mM S).

Analysis of S accumulation in *B. juncea* and *S. pinnata* in relation to S and Se supply (Two-Way ANOVA) showed a significant species effect (Factor A), a significant effect of S and Se dose (Factor B), as well as a significant interaction effect (*P* < 0.05) for the shoots (Tables 5S, 6S). Factors A and B also had significant effects on root S levels, but their interaction was not significant (Tables 7S, 8S). In *S. pinnata* plants, S accumulation in shoot and root was generally lower in S-starved plants than in S-replete plants (Figures 2C,D). Significant differences in *S*-values among plants supplied with different levels of S (0.5 or 5 mM) were observed only at 20 μM Se. The level of S was drastically reduced by Se in the shoot of both S-deplete plants and in plants replete with 0.5 mM S (Figure 2C). For example, addition of 10 μM selenate to 0.5 mM sulfate in the growth medium (1:50) reduced shoot S accumulation by 32%, from 11,000 to 7500 ug g^−1^ DW (Figure 2C). This effect was not evident in plants supplied with 5 mM S. Shoot S levels differed between the two plant species, depending on the treatment. In the absence of Se, *B. juncea* had the same S levels as *S. pinnata* under S-deplete conditions, lower S levels than *S. pinnata* under S-replete (0.5 mM S) conditions, and higher levels than *S. pinnata* under conditions of excess S (Figures 2A,C). In the presence of Se, *B. juncea* had higher S levels than *S. pinnata* except in 0.5 mM S, 10 μM Se, where S levels were the same. Sulfur accumulation in the roots of *S. pinnata* S-deficient plants was 2-fold reduced by 20 μM Se treatment (Figure 2D). The same Se treatment also diminished S levels in plants supplied with 0.5 S, but to a lesser extent. In the presence of 5 mM S, Se treatment did not affect S levels. Root S levels were overall lower in *S. pinnata* than *B. juncea*.

**Figure 3:**
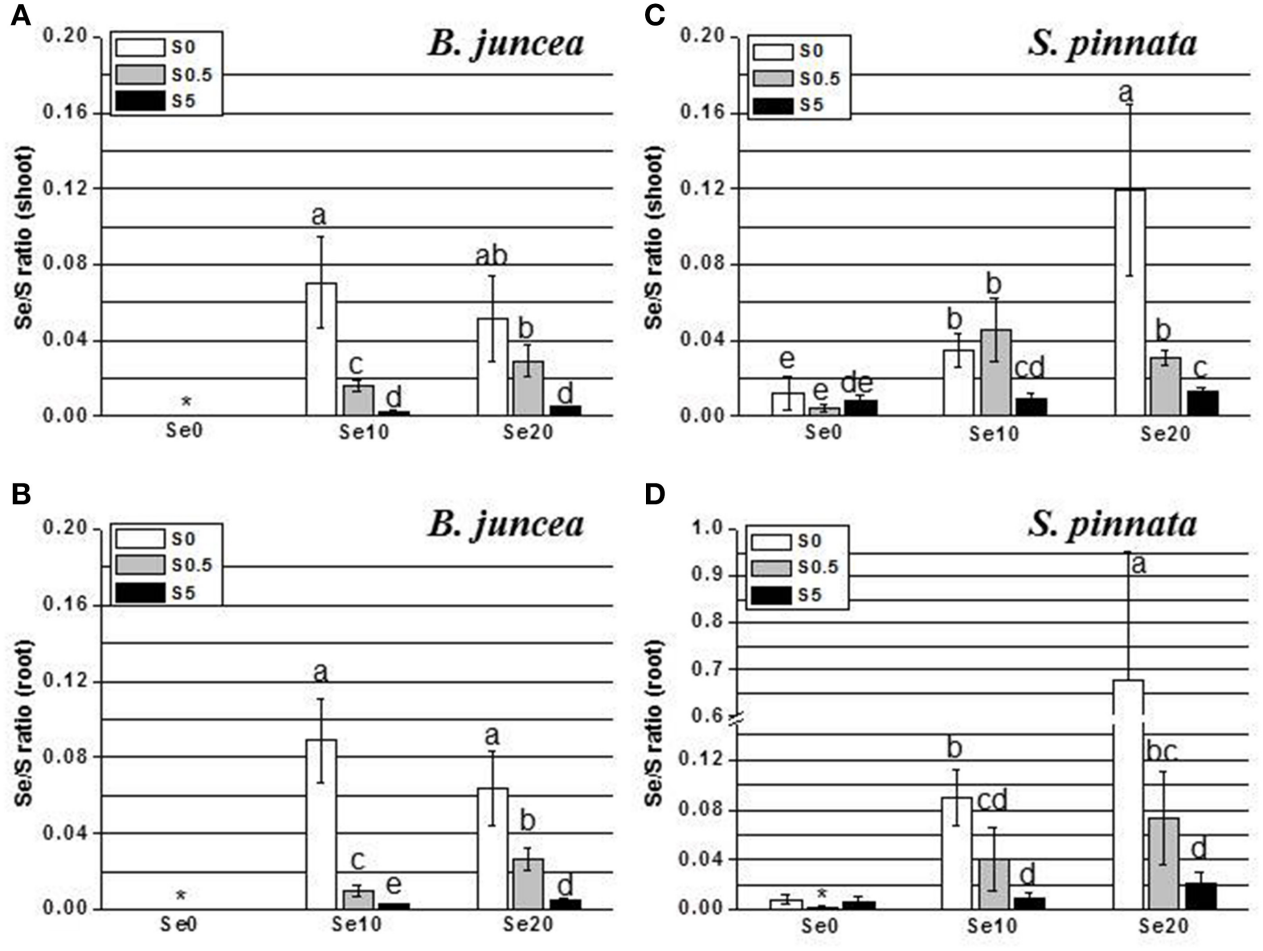
**Selenium:sulfur (Se/S) ratios in *B. juncea* and *S. pinnata* shoots (A,C, respectively) and roots (B,D, respectively) when of plants pretreated for 5 days in nutrient solution without sulfate and then supplied for 3 days with 0, 10, or 20 μM selenate and 0, 0.5, or 5 mM sulfate**. Note the scale difference. Data shown are the mean of five replicates ± SD. Letters above bars indicate significant differences between the means (*P* < 0.05). The asterisk indicate no significant differences among plants grown with different levels of sulfur in minus Se, and is referred to values significantly lower than those measured in Se-treated plants.

**Figure 4:**
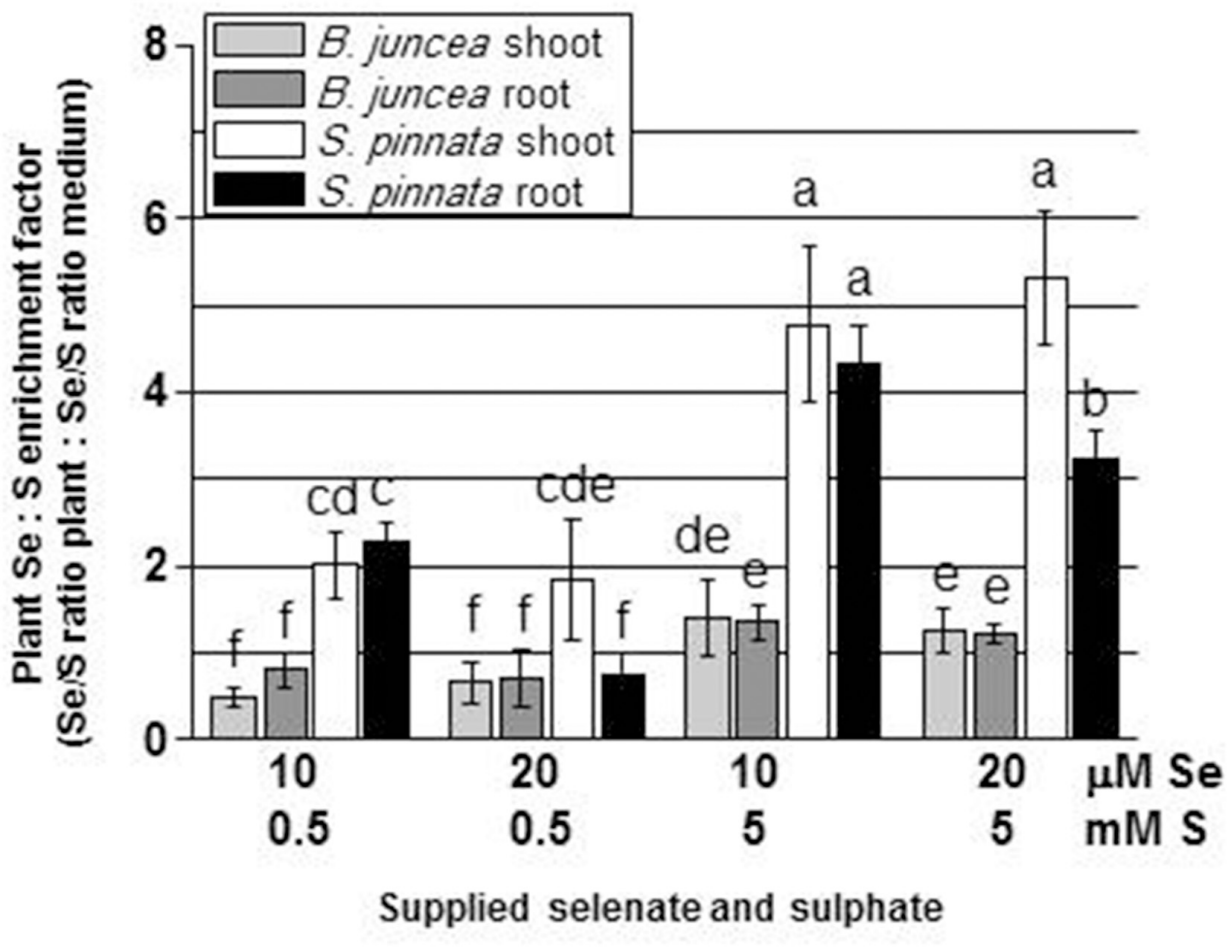
**Selenium enrichment relative to S in *B. juncea* and *S. pinnata* plants (calculated by dividing the Se/S ratio in the plant by the Se/S ratio in the growth medium)**. All plants were pretreated for 5 days in nutrient solution without sulfate and then supplied for 3 days with 0, 10, or 20 μM selenate and 0, 0.5, or 5 mM sulfate. Data shown are the mean of five replicates ± SD. Letters above bars indicate significant differences between the means (*P* < 0.05).

Tissue Se/S ratios were calculated, to obtain insight into how efficiently these elements competed for uptake into the two plant species. The results are shown in Figure 3 (note scale difference in panel D). Two-Way ANOVA of Se/S ratios in *B. juncea* and *S. pinnata* in relation to supplied S and Se indicated a significant species effect (Factor A), a significant effect of S and Se dose (Factor B), and a significant interaction effect (*P* < 0.05) for both the shoots (Tables 9S, 10S) and the roots (Tables 11S, 12S). In both species, the Se/S ratio decreased with S supply, as expected. For the majority of treatments, *S. pinnata* showed a higher tissue Se/S ratio than *B. juncea* in both the shoot and root (Figure 3). As a reference, the 10 μM selenate, 0.5 mM sulfate treatment had a Se/S ratio of 0.02 in the medium, and the 10 μM selenate, 5 mM sulfate treatment had a ratio of 0.002. Figure 4 shows plant Se/S ratio relative to supplied Se/S ratio, as a proxy for plant Se enrichment relative to S. Under normal S conditions (0.5 mM sulfate) *S. pinnata* plants showed 2-fold Se enrichment over S in their shoots at both the 10 and 20 μM selenate treatments, and also in roots at the 10 μM selenate treatment. In contrast, *B. juncea* plants from those same treatments did not show evidence of Se enrichment: their Se/S ratio was similar to, or lower than that of the medium (Figure 4). In the presence of excess S (5 mM sulfate), the difference in Se enrichment between the two plant species was even more profound. She Se/S ratio in *S. pinnata* root and shoot was 3.2- to 5.3-fold higher than that in the medium, while in *B. juncea* it was at most 1.3-fold that of the medium (Figure 4). These differences in Se/S ratio and Se enrichment factor between *S. pinnata* and *B. juncea* were significant even when seed-derived Se (i.e., plant Se concentration in the control plants) was subtracted from the plants given the +Se treatment (results not shown).

### Effects of different Se/S ratios on *Sultr1;1, Sultr1;2* and *Sultr2;1* gene expression in *B. juncea* and *S. pinnata*

The expression of group 1-sulfate transporters was assayed only in roots. *Sultr1;1* and *Sultr1;2* are mainly involved in the primary uptake of S/Se by roots. Based on the current literature, their expression in the shoot is usually undetectable under either normal S condition or short-term S-starvation (Buchner et al., 2004; Cabannes et al., 2011). On the other hand, the expression of *Sultr2;1* was evaluated in both root and leaf, because this transporter plays a pivotal role in sulfate loading/unloading in vascular tissues, and is commonly expressed at high level in both tissue types.

**Figure 5:**
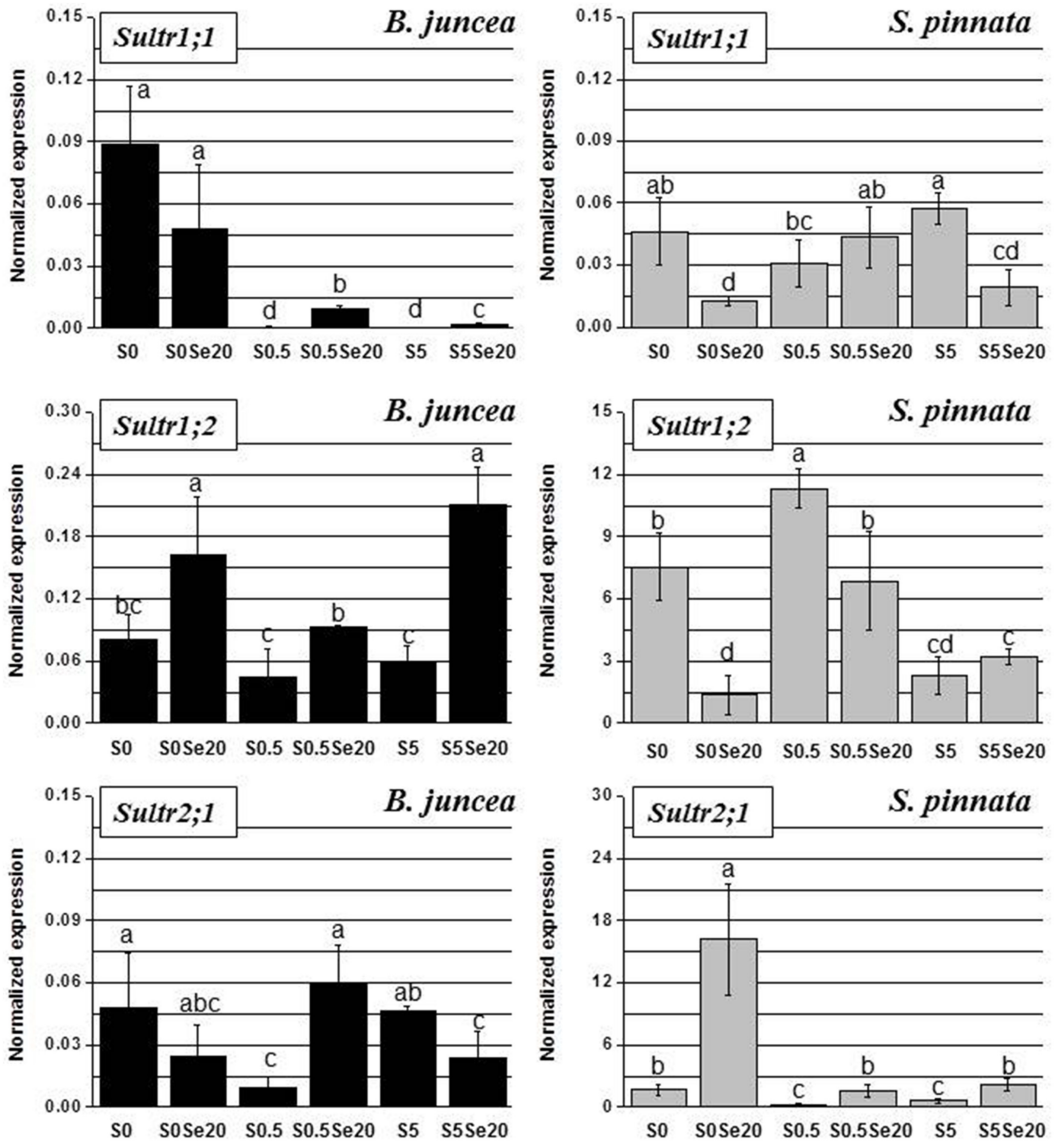
**Expression profiling by real-time RT-PCR of *Sultr1;1, Sultr1;2*, and *Sultr2;1* genes in roots of *B. juncea* and *S. pinnata* plants pretreated for 5 days in nutrient solution without sulfate and then supplied for 3 days with 0 or 20 μM selenate and 0, 0.5, or 5 mM sulfate**. Data shown are the mean ± SD of three replicates. Letters above bars indicate significant differences between the means (*P* < 0.05).

The two high-affinity sulfate transporters, *Sultr1;1* and *Sultr1;2*, as well as the low affinity transporter *Sultr2;1*, showed different root gene expression profiles in relation to S and Se supply in *B. juncea* than in *S. pinnata* (Figure 5). In addition, the two plant species showed vast differences in gene expression levels relative to each other. The transcript levels of sulfate transporter genes *Sultr1;2* and *Sultr2;1* were two orders of magnitude higher in *S. pinnata* than in *B. juncea*, as may be clear from a comparison of the Figure 5 y-axis scales. For instance, *Sultr1;2* was about 200-fold more expressed in *S. pinnata* plants supplied with 0.5 mM S and no Se (S0.5) than in *B. juncea* plants of the same treatment, and the *Sultr2;1* transcript level was 600-fold higher in *S. pinnata* S-deficient plants treated with Se (S0 Se20).

In *B. juncea*, the transcript level of *Sultr1;1* was much higher under S-deficiency, compared to plants provided with adequate or excess S amounts. While Se application did not significantly affect the expression of *Sultr1;1* in S-starved plants, it induced the transcript levels of this gene in S-sufficient plants. In *S. pinnata*, on the other hand, *Sultr1;1* was not up-regulated under S-deficiency, but *Sultr1;1* transcript was actually higher in plants supplied with 5 mM S than in plants grown with 0.5 mM S. The exposure of *S. pinnata* plants to Se caused repression of *Sultr1;1* transcription in minus S plants and in plants supplied with 5 mM S, and had no effect in plants provided with 0.5 mM S.

With respect to *Sultr1;2*, there were no clear effects of S supply on gene expression in *B. juncea*. However, we noted a ~2-fold upregulation under S-starvation in plants grown without Se. Regardless of S availability, Se-treated *B. juncea* plants had higher *Sultr1;2* transcript levels than their no-Se counterparts. In *S. pinnata, Sultr1;2* expression was highest in plants grown in the presence of 0.5 mM S, regardless of Se supply. The application of Se to *S. pinnata* plants was associated with 2–5-fold down-regulation of *Sultr1;2* in plants grown under S-limitation or in the presence of 0.5 mM S, while no effect was observed in plants provided with 5 mM S.

The *B. juncea* transcript levels of *Sultr2;1* were not clearly affected by S supply. Selenium-exposed plants showed 6-fold enhanced transcript levels of this transporter in plants grown with 0.5 mM S, but 2-fold lower *Sultr2;1* transcript levels at both other conditions of S supply. In *S. pinnata, Sultr2;1* transcript levels were clearly higher under S-limitation. The application of Se was associated with an increase in *Sultr2;1* transcript level, which was seen for all S treatments but most pronounced (8-fold) in S-starved plants.

**Figure 6:**
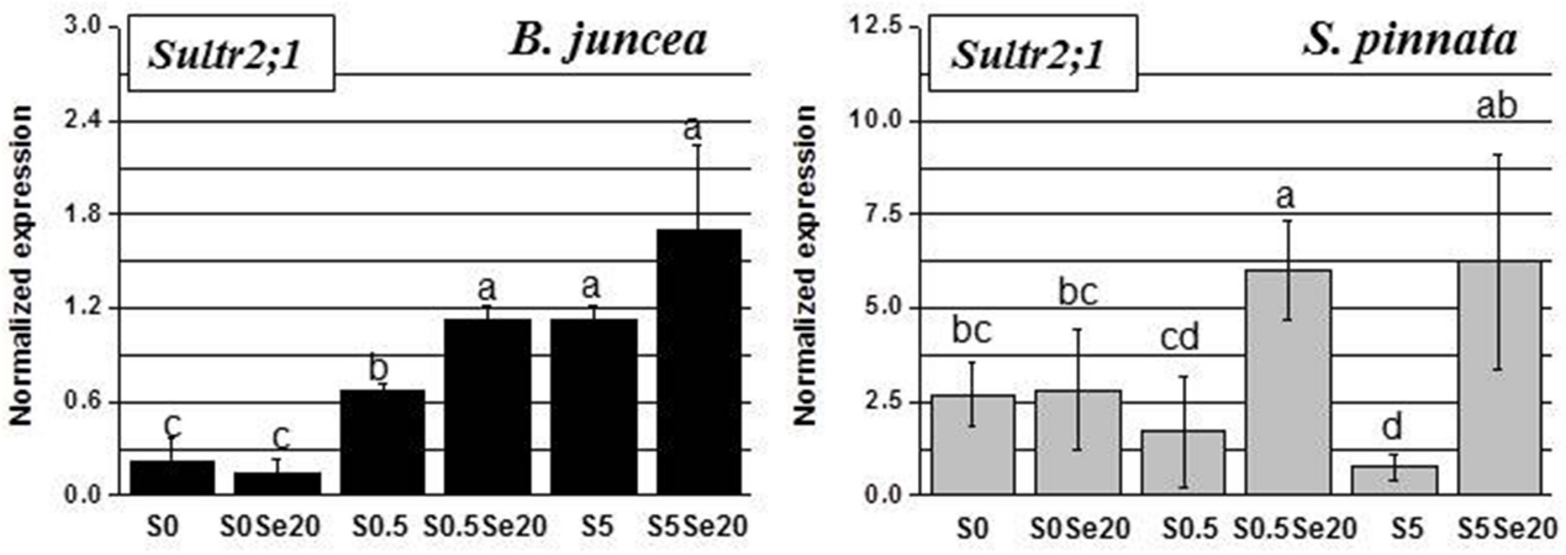
**Expression profiling by real-time RT-PCR of *Sultr2;1* gene in leaves of *B. juncea* and *S. pinnata* plants pretreated for 5 days in nutrient solution without sulfate and then supplied for 3 days with 0 or 20 μM selenate and 0, 0.5 or 5 mM sulfate**. Data shown are the mean ± SD of three replicates. Letters above bars indicate significant differences between the means (*P* < 0.05).

Leaf *Sultr2;1* expression showed opposite responses to S supply in the two plant species, when grown in the absence of Se. In *B. juncea* leaves, *Sultr2;1* transcript level went up with increasing S supply, while in *S. pinnata* the transcript level of *Sultr2;1* went down with S supply (Figure 6). Both species showed no Se effect on *Sultr2;1* expression in S-deficient plants, while Se treatment led to an increase in *Sultr2;1* transcript level in both species in S-replete plants. This effect was more pronounced in *S. pinnata* (4- to 6-fold) than in *B. juncea* (1.5–1.7-fold). It is noteworthy that the *Sultr2;1* transcript was generally more abundant in leaves of *S. pinnata* than in *B. juncea*, similar to what was found in the roots (see y-axis scales in Figure 6).

### Effects of different Se/S ratios on root and leaf *APS1, APS2* and *APS4* gene expression in *B. juncea* and *S. pinnata*

**Figure 7:**
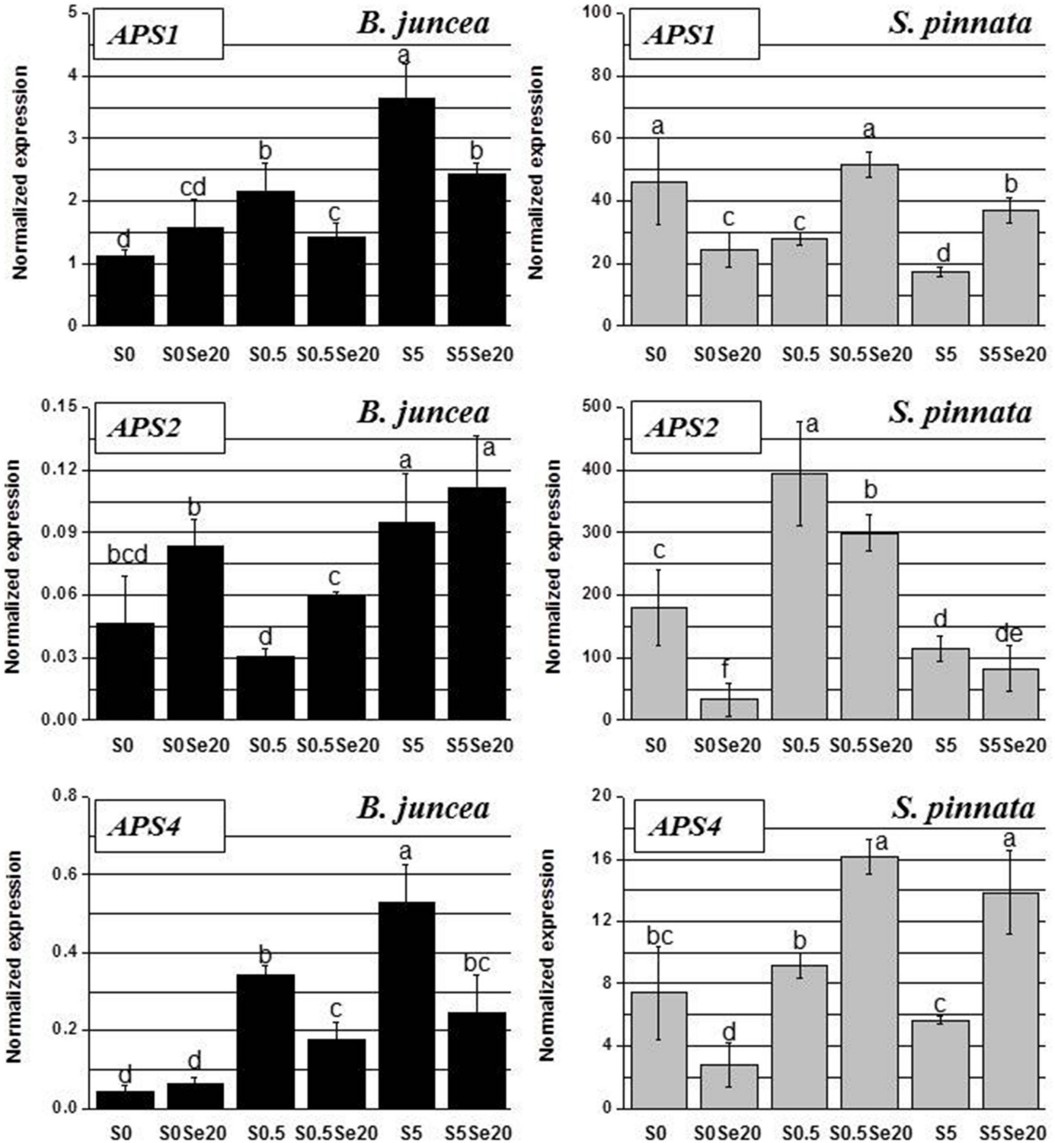
**Expression profiling by real-time RT-PCR of *APS1, APS2*, and *APS4* genes in roots of *B. juncea* and *S. pinnata* plants pretreated for 5 days in nutrient solution without sulfate and then supplied for 3 days with 0 or 20 μM selenate and 0, 0.5, or 5 mM sulfate**. Data shown are the mean ± SD of three replicates. Letters above bars indicate significant differences between the means (*P* < 0.05).

The root transcript levels and Se- and S-related patterns of APS isoforms displayed high variation between *B. juncea* and *S. pinnata* plants. In general, transcripts of all three genes, *APS1, APS2*, and *APS4*, were much more abundant in the hyperaccumulator, as it is apparent from the y-axis scales (Figure 7). The biggest difference (four orders of magnitude) was found for *APS2*, which was the least expressed isoform in *B. juncea* but the most highly expressed isoform in *S. pinnata*.

In *B. juncea*, the transcript accumulation of *APS1* and *APS4* were highly correlated (*R* = 0.93), while the expression of *APS2* gene followed a different trend. In *B. juncea* plants grown without Se, the transcript levels of *APS1* and *APS4* increased with S supply, while *APS2* expression did not show a clear S-related response. Selenium supply to S-starved *B. juncea* plants did not significantly affect *APS1* and *APS4* root transcript levels, while the transcript levels of both genes were reduced by Se in roots of S-sufficient and excess-S plants, after Se treatment. *APS2* transcript levels in *B. juncea* were generally up-regulated in the presence of Se, but this was only significant for the 0.5 mM S treatment.

In *S. pinnata* plants, too, the root transcript levels of *APS1* and *APS4* were correlated (*R* = 0.70), and *APS2* was regulated differently (Figure 7). Interestingly, opposite trends of transcript accumulation were observed between the two plant species for all of the *APS* genes. In *S. pinnata*, treatment with Se led to a reduction in *APS1* and *APS4* root transcript levels in S-starved plants, while it resulted in an increase in S-supplied plants. Transcript levels of *APS2* were reduced by Se in roots of *S. pinnata* under conditions of S-starvation or normal S levels; at excess S no significant effect was found. In roots of *S. pinnata* plants grown without Se, the *APS1* transcript level was highest under S-starvation and decreased with increasing S availability. Treatment with 0.5 mM S resulted in the highest transcript levels for *APS2* and *APS4*.

**Figure 8:**
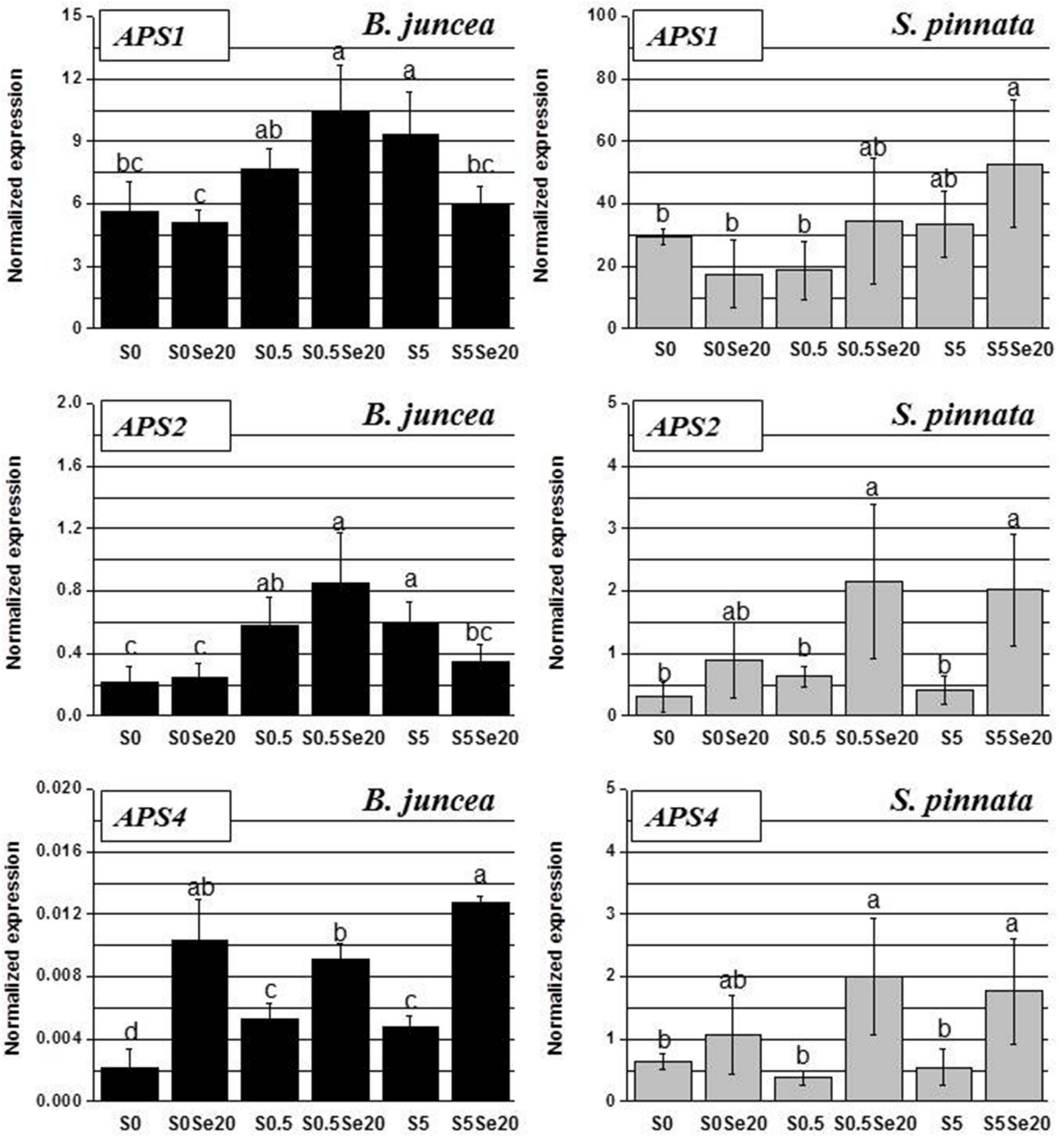
**Expression profiling by real-time RT-PCR of *APS1, APS2*, and *APS4* genes in shoots of *B. juncea* and *S. pinnata* plants pretreated for 5 days in nutrient solution without sulfate and then supplied for 3 days with 0 or 20 μM selenate and 0, 0.5 or 5 mM sulfate**. Data shown are the mean ± SD of three replicates. Letters above bars indicate significant differences between means (*P* < 0.05).

In leaves, as in roots, the trends of APS transcript accumulation in response to S and Se supply showed large variation between *B. juncea* and *S*. *pinnata* (Figure 8). The *APS* transcript levels in general were again higher for the hyperaccumulator, particularly those of *APS1* and *APS4* (compare y-axis scales). In leaves of both species, *APS1* was the most abundant transcript.

In *B. juncea* leaves, APS genes were generally more expressed in S-sufficient than in S-deplete plants, particularly when grown in the absence of Se. The leaf expression patterns of *APS1* and *APS2* strongly correlated (*R* = 0.97): Se treatment did not affect the transcription of these genes in S-starved plants, up-regulated it in plants supplied with 0.5 mM S and reduced it in plants grown in the presence of high S (5 mM). The transcript levels of *APS4* were consistently higher in Se-treated *B. juncea* plants, regardless of the external S availability.

**Figure 9:**
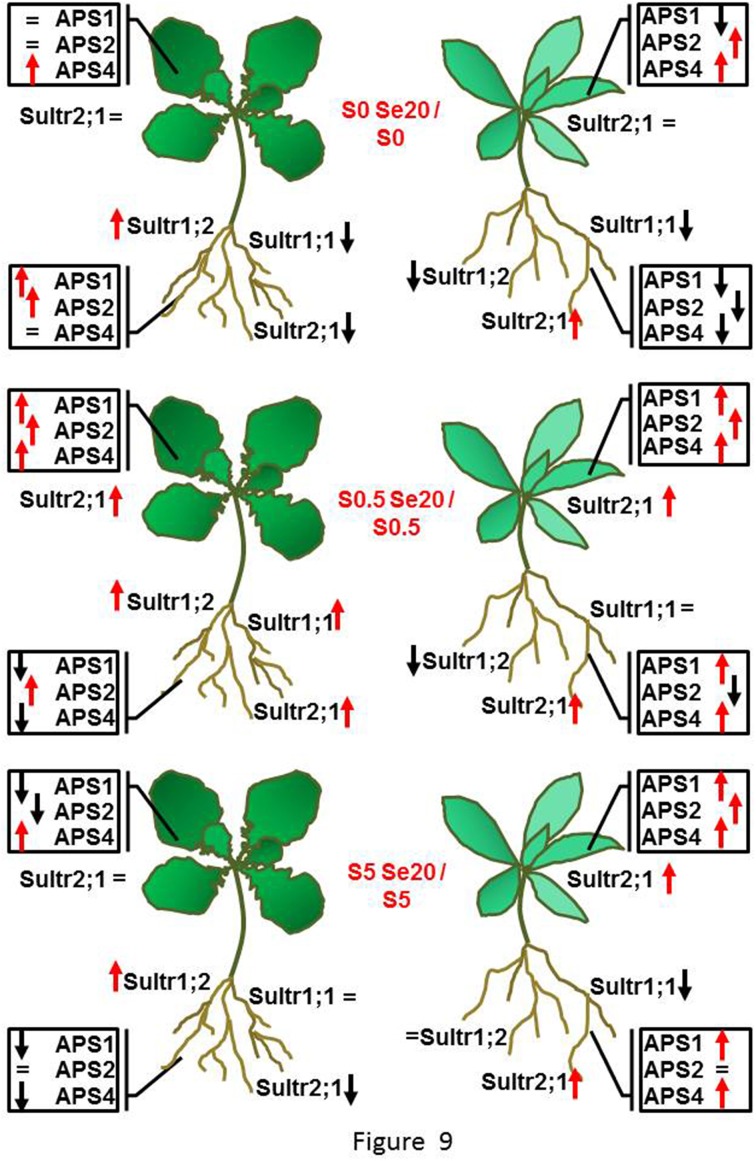
**Schematic diagram summarizing the responses in *B. juncea* (on the left side) and *S. pinnata* (on the right side) root and shoot *Sultr* and *APS* transcript levels to treatment with 20 μM selenate, at each of the three S supply levels (S-starved (no sulfate, top), S-replete (0.5 mM sulfate, middle), and excess S (5 mM sulfate, bottom)**. Red arrows indicate up-regulation, black arrows indicate down-regulation, = indicate no transcript variation, when expression was compared within each S treatment between plants treated with and without selenate.

In *S. pinnata* there was not as clear an effect of S on APS transcript levels as was found in *B. juncea*. There was a trend for APS expression to go down under S starvation but only in the presence of Se. In *S. pinnata* leaves, the trend of transcript accumulation was most similar between *APS2* and *APS4*, (*R* = 0.96); R was 0.60 between *APS1* and *APS2*, as well as between *APS1* and *APS4*. In general, the application of Se to plants stimulated the *APS* transcript levels in leaves of *S. pinnata*, except in S-starved plants. The differences in *B. juncea* and *S. pinnata Sultr* and *APS* expression patterns in response to Se and S are summarized in Figure 9.

## Discussion

The results from this study support the hypothesis that the Se hyperaccumulator *S. pinnata* has one or more root transporters with enhanced substrate specificity for selenate over sulfate, while the non-Se hyperaccumulator *B. juncea* does not show any evidence of discrimination between both substrates. Furthermore, *S. pinnata* showed evidence of highly elevated transcript levels for several sulfate/selenate transporters (*Sultr1;2* in roots, *Sultr2;1* in roots and shoots), as well as ATP sulfurylases (*APS2* in roots, *APS1* and *APS4* in roots and shoots), relative to *B. juncea*. These findings provide new insight into the mechanisms responsible for Se hyperaccumulation and hypertolerance in *S. pinnata*.

Despite being a well-documented Se hyperaccumulator (Freeman et al., 2006, 2010), in this study *S. pinnata* did not attain higher shoot Se levels than the secondary Se accumulator *B. juncea*; only in roots were the Se levels somewhat higher in *S. pinnata*. The hyperaccumulator had markedly higher Se/S ratios compared to *B. juncea*, which were due in large part to differences in S levels, particularly in the presence of Se. Selenate treatment reduced S levels in *S. pinnata*, while in *B. juncea* selenate promoted S accumulation, especially in the shoot. From the literature it is known that non-hyperaccumulators can respond to selenate treatment by increasing their sulfate uptake, which may be a mechanism to reduce Se toxicity in these species (Van Hoewyk et al., 2008; Harris et al., 2014). In Se hyperaccumulating *Astragalus* species, *A. racemosus* and *A. bisulcatus*, a Se-induced increase of shoot sulfate accumulation was also observed (Cabannes et al., 2011). Therefore, the reduction in S level in response to selenate treatment in *S. pinnata* is rather unusual. It may point to out-competition of sulfate by selenate during root membrane transport, if a primary *S. pinnata* sulfate/selenate transporter has higher specificity for selenate. The finding that Se accumulation in *S. pinnata* was much less responsive to external sulfate supply than *B. juncea* (2–3-fold rather than 10-fold) also points to enhanced selenate-specificity of a *S. pinnata* sulfate/selenate transporter. The explanation for the finding that there was still 2–3-fold inhibition of selenate uptake when sulfate was supplied in excess (two orders of magnitude higher levels of sulfate than selenate) may be that in *S. pinnata*, transporters with elevated specificity for selenate can still transport sulfate to some degree, and this is especially visible when sulfate is present at much higher concentration than selenate. Additionally, there are multiple SULTR proteins in the root plasma membrane that may differ in selenate specificity in *S. pinnata*, and in S-dependent expression level.

The analysis of *Sultr* gene expression revealed extraordinary accumulation of two SULTR transcripts in *S. pinnata* compared to *B. juncea*: high-affinity transporter SULTR1;2 and low-affinity transporter SULTR2;1. The former is thought to be the main portal for sulfate and selenate into the root, while the latter is responsible for translocation from root to shoot via the vasculature (Takahashi et al., 2011). High-affinity transporter SULTR1;1, thought to be of secondary importance for uptake into the root (Barberon et al., 2008), did not show much difference in overall expression level between the plant species. The finding that SULTR1;2 and SULTR2;1 are overexpressed in *S. pinnata* may explain earlier findings that this Se hyperaccumulator accumulates much higher levels of Se compared to related non-hyperaccumulators, especially in its shoot (Galeas et al., 2007; El Mehdawi et al., 2012; Harris et al., 2014). However, it cannot readily be explained why the enhanced transcript levels did not correspond with much higher Se levels in this particular study. Only in roots were Se levels somewhat higher in *S. pinnata* than *B. juncea*. Perhaps there is another tier of regulation, at the protein level, that moderates the extraordinary transcript levels. SULTR1;2 has been reported in *A. thaliana* to be feed-back inhibited via interaction of a C-terminal STAS domain with a cytosolic cysteine synthase (Shibagaki and Grossman, 2010); a similar mechanism may exist in *S. pinnata*.

In addition to overall *Sultr* expression level differences, the two plant species differed in their Se- and S-related responses. In *B. juncea, Sultr1;1* appeared to be nearly totally repressed under sufficient S supply, while its expression was strongly induced under S starvation. This was not at all observed in *S. pinnata*. *Sultr1;2* was not affected by S supply in either species. The finding that *Sultr1;1* was upregulated by S starvation in *B. juncea*, while *Sultr1;2* was not, is in agreement with previous studies (Yoshimoto et al., 2002; Rouached et al., 2008). *Sultr1;1* and *Sultr1;2* were upregulated by selenate treatment in S-sufficient *B. juncea* plants, which may explain the observed increase in S (and Se) accumulation in this species. In *S. pinnata, Sultr1;1* and *Sultr1;2* were not upregulated by Se treatment, and their transcript levels were even repressed in selenate-treated plants grown under S-deficient conditions. The down-regulation by Se of these high-affinity sulfate transporters under conditions of S starvation may be envisioned as a Se-tolerance mechanism to reduce the entry of excessive Se when sulfate is not available for uptake, especially if one or more transporters have higher selectivity for selenate over sulfate and considering how high these transcript levels are compared to *B. juncea*. The reduced S compound glutathione may play a role in Se tolerance in *S. pinnata*, as it may mediate non-enzymatic selenite reduction (Terry et al., 2000) or via formation of selenodiglutathione (Freeman et al., 2010). When sulfate was available at sufficient levels, this effect of Se on transcript levels of these high-affinity transporters in *S. pinnata* was much less pronounced or absent. The finding that *Sultr1;1* expression was not S-dependent in *S. pinnata* is similar to previous findings in the Se-hyperaccumulators *A. racemosus* and *A. bisulcatus*, where the transcript abundance of *Sultr1;1* occurred at a high level even in the presence of external S (Cabannes et al., 2011). It may be a common property of Se hyperaccumulating species to have a high potential sulfate uptake capacity, irrespective of sulfate supply, which facilitates high selenate uptake regardless of external S levels.

*Stanleya pinnata* accumulated much higher transcript levels of all three *APS* genes tested, compared to *B. juncea*. This was particularly striking for *APS2* in the root and *APS4* in the shoot, where transcript levels were 2–3 orders of magnitude higher in the hyperaccumulator. Previous work showed that ATP sulfurylase not only mediates selenate reduction in plants, but is also a rate limiting enzyme for selenate uptake and assimilation (Pilon-Smits et al., 1999). Overexpression of *A. thaliana APS1* in *B. juncea* was found to enhanced Se accumulation, reduction and tolerance (Pilon-Smits et al., 1999). If the enhanced *APS* transcript levels observed here in *S. pinnata* correlate with enhanced levels of the corresponding enzyme activity, and if this activity is also limiting for selenate assimilation in *S. pinnata*, then the assimilation of selenate to organic selenocompounds likely occurs more efficiently in this hyperaccumulator. Indeed, the main forms of Se in this species, both in the field and when supplied with selenate in controlled studies, have been reported to be methyl-selenocysteine and selenocystathionine (Freeman et al., 2006). Since these compounds are not specifically incorporated into proteins and therefore do not disrupt protein function, the ability to accumulate Se in these organic forms is considered a key mechanism for Se hypertolerance (Neuhierl and Böck, 2002; Freeman et al., 2010). *Brassica juncea* accumulates mainly selenate in such conditions, but when genetically engineered to overexpress *APS1*, it accumulated organic Se (Pilon-Smits et al., 1999). These results agree with those from Se hyperaccumulating *Astragalus* species, where APS enzymes have been identified as major contributors of Se reduction in plants, and the Se hyperaccumulation trait was proposed to be driven by an increased Se flux through the S assimilatory pathway generated by Se-organic compounds (Cabannes et al., 2011). Therefore, it is reasonable to hypothesize that the elevated expression of *APS* isoform genes we observed in *S. pinnata* is a key mechanism for their ability to hyperaccumulate and hypertolerate Se. It is interesting to note that *S. pinnata* showed extraordinarily high expression of *APS2* compared other *APS* isoforms in its roots, which may indicate that APS2 is the key enzyme for Se assimilation into organic forms in this species, and that the roots play an important role in this process. More studies are needed to investigate this hypothesis.

There were some interesting differences between the plant species with respect to APS transcript responses to S and Se supply. In contrast to *B. juncea, S. pinnata* showed down-regulation of all three *APS* genes in roots of S-deficient plants in response to Se treatment. Similar responses were observed for the high-affinity *Sultr* genes. As mentioned, this may serve to reduce excessive Se accumulation in tissues, especially in consideration of the abundance of *Sultr* and *APS* transcripts. While APS contributes to Se tolerance by being a key enzyme for the conversion to non-toxic organic forms, some of the intermediates, such as selenite or selenocysteine, may cause toxicity if they accumulate. This downregulation in the hyperaccumulator may represent a tolerance mechanism to Se in the absence of S, which is not present in the non-hyperaccumulator *B. juncea*. When S was not limiting, Se did not affect *APS2* transcript levels in *S. pinnata*, and actually resulted in transcript up-regulation of *APS1* and *APS4*. The divergence in the gene expression patterns between *APS2* on the one side and *APS1* and *APS4* on the other, was generally observed in both plant species, and may be due to different types of regulatory mechanisms and subcellular localization. *APS1* and *APS4* are known to be subjected to post-transcriptional regulation mediated by miRNA395 (Kawashima et al., 2009; Liang and Yu, 2010), while *APS2* is not. Furthermore, *APS1* and *APS4* encode isoforms that are only plastidic (Leustek et al., 1994; Hatzfeld et al., 2000), while APS2 may colocalize to both the plastids and the cytosol.

## Conclusions

To date, no specific selenate transporter has been identified in any organism, although its existence has been hypothesized in Se hyperaccumulators. The results obtained in this study support the hypothesis that the Se-hyperaccumulator *S. pinnata* possesses at least one transporter with elevated selenate specificity over sulfate in comparison to *B. juncea*. Further transgenic experiments are needed to identify this/these putative selenate transporter(s), as well as kinetic experiments to study the properties and S/Se discriminatory mechanisms of putative selenate transporters in *S. pinnata*. *S. pinnata* was found here to have a significantly higher transcript expression level of *Sultr1;2*, thought to be the main transporter for selenate uptake into roots, as well as of *Sultr2;1*, responsible for selenate translocation to the shoot. These genes will be good candidates for further studies. The observed vastly higher expression levels in *S. pinnata* of several *APS* genes, involved in conversion of selenate to non-toxic organic selenocompounds, likely contributes to the Se hypertolerance of this species.

The findings presented here have relevance for both Se phytoremediation and biofortification. Both technologies are hindered by high S levels, suboptimal plant Se accumulation or Se phytotoxicity. The identification of a selenate-specific transporter could be used to generate crops with selenate-specific uptake in high-S environments. Also, the *APS* genes found to be upregulated here may be used to enhance plant Se tolerance though more efficient conversion of inorganic selenate to less toxic organic forms of Se. These processes also have relevance for medicine. Selenate transporters may be expressed in other organisms such as bacteria or yeast, and insight into selenate/sulfate discrimination mechanisms may be used to manipulate substrate specificity of other proteins. Also, since organic selenocompounds are more suitable for animal nutrition than inorganic forms, and may even have anti-carcinogenic properties (Hatfield et al., 2014), better ways to convert inorganic to organic Se in organisms used for the production of Se supplements, e.g., via the use of a highly active APS enzyme, may benefit human health.

### Conflict of interest statement

The authors declare that the research was conducted in the absence of any commercial or financial relationships that could be construed as a potential conflict of interest.
