# Supplementary material for: Exploring the importance of sulfate transporters and ATP sulphurylases for selenium hyperaccumulation—a comparison of Stanleya pinnata and Brassica juncea (Brassicaceae)
Source: Front Plant Sci. 2015 Jan 23;6:2. doi: 10.3389/fpls.2015.00002 (PMC4304243; doi:10.3389/fpls.2015.00002)
Supplement: Supplementary file 1 [file Table1.DOCX]

**Table 1S. Two-way ANOVA analysis of Se accumulation in the shoot of *B. juncea* and *S. pinnata* in relation to the S and Se dose applied. Analysis of Factor A (species) and Factor B (S and Se dose) interaction was additionally performed.**

|  | DF | Sum of Squares | Mean Square | F Value | P Value |
| --- | --- | --- | --- | --- | --- |
| FactorA | 1 | 26295.96402 | 26295.96 | 7.37426 | 0.0101 |
| FactorB | 8 | 1.00E+06 | 125246.1 | 35.12315 | 9.33E-15 |
| Interaction | 8 | 335045.3564 | 41880.67 | 11.74472 | 4.93E-08 |
| Model | 17 | 1.36E+06 | 80194.73 | 22.48925 | 3.11E-14 |
| Error | 36 | 128372.9178 | 3565.914 | -- | -- |
| Corrected Total | 53 | 1.49E+06 | -- | -- | -- |
| **Factor A= species** |  |  |  |  |  |
| **Factor B= S and S doses** |  |  |  |  |  |
| At the 0.05 level. the population means of Factor A are significantly different | | | | | |
| At the 0.05 level. the population means of Factor B are significantly different | | | | | |
| At the 0.05 level. the interaction between Factor A and Factor B is significant | | | | | |

**Table 2S. Comparison between species in shoot Se accumulation in relation to the S-Se dose applied. Values in bold indicate significant differences for P<0.05.**

|  | ***B. juncea*** | | | | | | | | | |
| --- | --- | --- | --- | --- | --- | --- | --- | --- | --- | --- |
|  |  | *S0Se0* | *S0Se10* | *S0Se20* | *S0.5Se0* | *S0.5Se10* | *S0.5Se20* | *S5Se0* | *S5Se10* | *S5Se20* |
| ***S. pinnata*** | *S0Se0* | 0.98204 | **7.29E-05** | 0 | 0.98057 | 0.99919 | **0.04342** | 0.98095 | 1 | 1 |
|  | *S0Se10* | 0.42881 | **0.00217** | **5.74E-08** | 0.42076 | 1 | 0.44037 | 0.4228 | 0.88789 | 0.99778 |
|  | *S0Se20* | **5.29E-04** | 0.7445 | **2.32E-07** | **5.09E-04** | 0.4022 | 1 | **5.14E-04** | **0.00482** | **0.02875** |
|  | *S0.5Se0* | 0.99995 | **1.17E-05** | **2.14E-08** | 0.99994 | 0.94034 | **0.00884** | 0.99995 | 1 | 1 |
|  | *S0.5Se10* | **0.0234** | 0.08199 | **1.78E-07** | **0.02263** | 0.99381 | 0.997 | **0.02283** | 0.14303 | 0.46218 |
|  | *S0.5Se20* | 0.09046 | **0.02092** | **1.07E-06** | 0.08785 | 0.99999 | 0.91562 | 0.0885 | 0.39393 | 0.81454 |
|  | *S5Se0* | 0.99358 | **4.64E-05** | 0 | 0.99293 | 0.99662 | **0.02977** | 0.9931 | 1 | 1 |
|  | *S5Se10* | 0.9982 | **2.96E-05** | 0 | 0.99797 | 0.98932 | **0.02018** | 0.099803 | 1 | 1 |
|  | *S5Se20* | 0.058601 | **0.00108** | **5.65E-08** | 0.57732 | 1 | 0.30178 | 0.57952 | 0.95957 | 0.99983 |

**Table 3S. Two-way ANOVA analysis of Se accumulation in roots of *B. juncea* and *S. pinnata* in relation to the S and Se dose applied. Analysis of Factor A (species) and Factor B (treatment) interaction was additionally performed.**

|  | DF | Sum of Squares | Mean Square | F Value | P Value |
| --- | --- | --- | --- | --- | --- |
| FactorA | 1 | 30556.35207 | 30556.35 | 6.16198 | 0.01785 |
| FactorB | 8 | 1.25E+06 | 156227.6 | 31.50477 | 5.05E-14 |
| Interaction | 8 | 111822.2232 | 13977.78 | 2.81875 | 0.01553 |
| Model | 17 | 1.39E+06 | 81894.09 | 16.51472 | 3.74E-12 |
| Error | 36 | 178518.7986 | 4958.856 | -- | -- |
| Corrected Total | 53 | 1.57E+06 | -- | -- | -- |
| **Factor A= species** |  |  |  |  |  |
| **Factor B= S and S doses** |  |  |  |  |  |
| At the 0.05 level. the population means of Factor A are significantly different | | | | |  |
| At the 0.05 level. the population means of Factor B are significantly different | | | | |  |
| At the 0.05 level. the interaction between Factor A and Factor B is significant | | | | |  |

**Table 4S. Comparison between species in root Se accumulation in relation to the S-Se dose applied. Values in bold indicate significant differences for P<0.05.**

|  | ***B. juncea*** | | | | | | | | | |
| --- | --- | --- | --- | --- | --- | --- | --- | --- | --- | --- |
|  |  | *S0Se0* | *S0Se10* | *S0Se20* | *S0.5Se0* | *S0.5Se10* | *S0.5Se20* | *S5Se0* | *S5Se10* | *S5Se20* |
| ***S. pinnata*** | *S0Se0* | 0.590 | **3.86E-07** | **0.00152** | 1 | 0.9987 | **0.02483** | 1 | 1 | 1 |
|  | *S0Se10* | **6.20E-04** | 0.28121 | 1 | **6.80E-04** | **0.05208** | 0.99997 | **6.20E-04** | **0.0022** | **0.00801** |
|  | *S0Se20* | **1.21E-07** | 1 | 0.2403 | **1.26E-07** | **6.48E-06** | **0.02446** | **1.21E-07** | **2.57E-07** | **7.74E-07** |
|  | *S0.5Se0* | 1 | **4.51E-07** | **0.00183** | 1 | 0.99933 | **0.02924** | 1 | 1 | 1 |
|  | *S0.5Se10* | **0.03488** | **0.00954** | 0.97845 | **0.03776** | 0.68832 | 1 | **0.03488** | 0.09835 | 0.25079 |
|  | *S0.5Se20* | **2.67E-04** | 0.44304 | 1 | **2.94E-04** | **0.02558** | 0.99884 | **2.67E-04** | **9.65E-04** | **0.00362** |
|  | *S5Se0* | 0.99999 | **1.14E-06** | **0.00494** | 0.99999 | 0.99999 | 0.06868 | 0.9999 | 1 | 1 |
|  | *S5Se10* | 0.99997 | **1.37E-06** | **0.0059** | 0.99998 | 1 | 0.079637 | 0.9997 | 1 | 1 |
|  | *S5Se20* | 0.45783 | **2.49E-04** | 0.34239 | 0.47829 | 0.99981 | 0.92359 | 0.45783 | 0.74287 | 0.94496 |

**Table 5S. Two-way ANOVA of S accumulation in the shoot of *B. juncea* and *S. pinnata* in relation to the S and Se dose applied. Analysis of Factor A (species) and Factor B (treatment) interaction was additionally performed.**

|  | DF | Sum of Squares | Mean Square | F Value | P Value |
| --- | --- | --- | --- | --- | --- |
| FactorA | 1 | 9.72E+07 | 9.72E+07 | 44.13673 | 9.68E-08 |
| FactorB | 8 | 3.83E+08 | 4.79E+07 | 21.72905 | 1.30E-11 |
| Interaction | 8 | 1.11E+08 | 1.39E+07 | 6.30318 | 4.22E-05 |
| Model | 17 | 5.91E+08 | 3.48E+07 | 15.78791 | 7.37E-12 |
| Error | 36 | 7.93E+07 | 2.20E+06 | -- | -- |
| Corrected Total | 53 | 6.70E+08 | -- | -- | -- |
| **Factor A= species** |  |  |  |  |  |
| **Factor B= S and S doses** |  |  |  |  |  |
| At the 0.05 level. the population means of Factor A are significantly different | | | | |  |
| At the 0.05 level. the population means of Factor B are significantly different | | | | |  |
| At the 0.05 level. the interaction between Factor A and Factor B is significant | | | | |  |

**Table 6S. Comparison between species in shoot S accumulation in relation to the S-Se dose applied. Values in bold indicate significant differences for P<0.05.**

|  | ***B. juncea*** | | | | | | | | | |
| --- | --- | --- | --- | --- | --- | --- | --- | --- | --- | --- |
|  |  | *S0Se0* | *S0Se10* | *S0Se20* | *S0.5Se0* | *S0.5Se10* | *S0.5Se20* | *S5Se0* | *S5Se10* | *S5Se20* |
| ***S. pinnata*** | *S0Se0* | 1 | 0.99998 | 1 | 0.95996 | 0.97839 | 0.66986 | **0.00917** | **1.11E-05** | **9.75E-06** |
|  | *S0Se10* | 0.6015 | 0.91787 | 0.58967 | **0.00997** | **0.01354** | **0.00164** | **3.26E-06** | **1.77E-07** | **1.78E-07** |
|  | *S0Se20* | 0.12844 | 0.38118 | 0.12367 | **7.16E-04** | **9.98E-04** | **1.07E-04** | **2.68E-07** | **5.94E-08** | **5.92E-08** |
|  | *S0.5Se0* | **0.02047** | **0.00417** | **0.02143** | 0.77299 | 0.70412 | 0.98438 | 1 | 0.31576 | 0.29367 |
|  | *S0.5Se10* | 0.99927 | 0.94932 | 0.9994 | 0.99999 | 1 | 0.98866 | 0.08059 | **1.44E-04** | **1.27E-04** |
|  | *S0.5Se20* | 1 | 1 | 1 | 0.66193 | 0.73406 | 0.26901 | **0.00145** | **1.61E-06** | **1.40E-06** |
|  | *S5Se0* | 0.98463 | 0.80594 | 0.98628 | 1 | 1 | 0.99956 | 0.17768 | **4.27E-04** | **3.77E-04** |
|  | *S5Se10* | 1 | 0.99757 | 1 | 0.99715 | 0.99902 | 0.8861 | **0.02613** | **3.62E-05** | **3.18E-05** |
|  | *S5Se20* | 0.25395 | 0.0751 | 0.26219 | 0.99977 | 0.99915 | 1 | 0.95624 | **0.02822** | **0.02531** |

**Table 7S. Two-way ANOVA of S accumulation in roots of *B. juncea* and *S. pinnata* in relation to the S and Se dose applied. Analysis of Factor A (species) and Factor B (treatment) interaction was additionally performed.**

|  | DF | Sum of Squares | Mean Square | F Value | P Value |
| --- | --- | --- | --- | --- | --- |
| FactorA | 1 | 2.09E+08 | 2.09E+08 | 60.10688 | 4.43E-11 |
| FactorB | 8 | 2.46E+08 | 3.08E+07 | 8.84441 | 2.41E-08 |
| Interaction | 8 | 3.09E+07 | 3.86E+06 | 1.10913 | 0.36745 |
| Model | 17 | 4.86E+08 | 2.86E+07 | 8.21972 | 5.16E-11 |
| Error | 72 | 2.50E+08 | 3.48E+06 | -- | -- |
| Corrected Total | 89 | 7.37E+08 | -- | -- | -- |
| **Factor A= species** |  |  |  |  |  |
| **Factor B= S and S doses** |  |  |  |  |  |
| At the 0.05 level. the population means of Factor A are significantly different | | | | |  |
| At the 0.05 level. the population means of Factor B are significantly different | | | | |  |
| At the 0.05 level. the interaction between Factor A and Factor B is not significant | | | | |  |

**Table 8S. Comparison between species in root S accumulation in relation to the S-Se dose applied. Values in bold indicate significant differences for P<0.05.**

|  | ***B. juncea*** | | | | | | | | | |
| --- | --- | --- | --- | --- | --- | --- | --- | --- | --- | --- |
|  |  | *S0Se0* | *S0Se10* | *S0Se20* | *S0.5Se0* | *S0.5Se10* | *S0.5Se20* | *S5Se0* | *S5Se10* | *S5Se20* |
| ***S. pinnata*** | *S0Se0* | 0.99942 | 0.99904 | 0.99828 | **0.01161** | **3.26E-04** | **0.00103** | **0.00297** | **0.00263** | **2.89E-04** |
|  | *S0Se10* | 0.99978 | 0.99961 | 0.99926 | **0.00664** | **1.70E-04** | **5.47E-04** | **0.00163** | **0.00144** | **1.50E-04** |
|  | *S0Se20* | 0.32959 | 0.32027 | 0.28571 | **3.67E-05** | **6.25E-07** | **2.13E-06** | **7.16E-06** | **6.21E-06** | **5.54E-07** |
|  | *S0.5Se0* | 0.99997 | 0.99998 | 0.99999 | 0.8766 | 0.2376 | 0.41928 | 0.63126 | 0.60621 | 0.2227 |
|  | *S0.5Se10* | 1 | 1 | 1 | 0.22792 | **0.01324** | **0.03545** | 0.08392 | 0.07625 | **0.01194** |
|  | *S0.5Se20* | 1 | 0.99999 | 0.99998 | 0.77879 | **0.00548** | **0.01420** | **0.03353** | **0.03042** | **0.00496** |
|  | *S5Se0* | 0.99999 | 0.99242 | 0.99526 | 0.99298 | 0.57957 | 0.78850 | 0.92595 | 0.91418 | 0.55708 |
|  | *S5Se10* | 1 | 1 | 1 | 0.28942 | **0.0221** | 0.05438 | 0.1187 | 0.10888 | **0.02009** |
|  | *S5Se20* | 1 | 1 | 1 | 1 | 0.84857 | 0.96801 | 0.99783 | 0.99821 | 0.98261 |

**Table 9S. Two-way ANOVA of Se/S ratio in the shoot of *B. juncea* and *S. pinnata* in relation to the S and Se dose applied. Analysis of Factor A (species) and Factor B (treatment) interaction was additionally performed.**

|  | DF | Sum of Squares | Mean Square | F Value | P Value |
| --- | --- | --- | --- | --- | --- |
| FactorA | 1 | 30556.35 | 30556.35 | 6.16198 | 0.01785 |
| FactorB | 8 | 1.25E+06 | 156227.6 | 31.50477 | 5.05E-14 |
| Interaction | 8 | 111822.2 | 13977.78 | 2.81875 | 0.01553 |
| Model | 17 | 1.39E+06 | 81894.09 | 16.51472 | 3.74E-12 |
| Error | 36 | 178518.8 | 4958.856 | -- | -- |
| Corrected Total | 53 | 1.57E+06 | -- | -- | -- |
| **Factor A= species** |  |  |  |  |  |
| **Factor B= S and S doses** |  |  |  |  |  |
| At the 0.05 level. the population means of Factor A are significantly different | | | | |  |
| At the 0.05 level. the population means of Factor B are significantly different | | | | |  |
| At the 0.05 level. the interaction between Factor A and Factor B is significant | | | | |  |

**Table 10S. Comparison between species in shoot Se/S ratio in relation to the S-Se dose applied. Values in bold indicate significant differences for P<0.05.**

|  | ***B. juncea*** | | | | | | | | | |
| --- | --- | --- | --- | --- | --- | --- | --- | --- | --- | --- |
|  |  | *S0Se0* | *S0Se10* | *S0Se20* | *S0.5Se0* | *S0.5Se10* | *S0.5Se20* | *S5Se0* | *S5Se10* | *S5Se20* |
| ***S. pinnata*** | *S0Se0* | 0.99998 | **0.00606** | 0.27485 | 0.99998 | 1 | 0.99864 | 0.99998 | 1 | 1 |
|  | *S0Se10* | 0.50553 | 0.42883 | 0.99825 | 0.50126 | 0.99578 | 1 | 0.50108 | 0.64256 | 0.75128 |
|  | *S0Se20* | **2.06E-09** | **0.05368** | **5.37E-04** | **2.04E-09** | **4.99E-08** | **7.34E-07** | **2.04E-09** | **2.76E-09** | 0 |
|  | *S0.5Se0* | 1 | **6.96E-04** | 0.06502 | 1 | 0.99998 | 0.92056 | 1 | 1 | 1 |
|  | *S0.5Se10* | 0.0979 | 0.82548 | 1 | 0.09636 | 0.79134 | 0.99949 | 0.09629 | 0.15855 | 0.22906 |
|  | *S0.5Se20* | 0.84561 | 0.29200 | 0.98221 | 0.84275 | 0.99997 | 1 | 0.84262 | 0.92106 | 0.96133 |
|  | *S5Se0* | 1 | **0.00950** | 0.13443 | 1 | 1 | 0.98184 | 1 | 1 | 1 |
|  | *S5Se10* | 1 | **0.00246** | 0.15664 | 1 | 1 | 0.98815 | 1 | 1 | 1 |
|  | *S5Se20* | 0.99995 | **0.00719** | 0.30319 | 0.99995 | 1 | 0.99919 | 0.99995 | 1 | 1 |

**Table 11S. Two-way ANOVA of Se/S ratio in the root of *B. juncea* and *S. pinnata* in relation to the S and Se dose applied. Analysis of Factor A (species) and Factor B (treatment) interaction was additionally performed.**

|  | DF | Sum of Squares | Mean Square | F Value | P Value |
| --- | --- | --- | --- | --- | --- |
| FactorA | 1 | 0.15028 | 0.15028 | 7.41778 | 0.0081 |
| FactorB | 8 | 1.14514 | 0.14314 | 7.06542 | 7.53E-07 |
| Interaction | 8 | 0.81042 | 0.1013 | 5.00025 | 5.97E-05 |
| Model | 17 | 2.10584 | 0.12387 | 6.1143 | 1.84E-08 |
| Error | 72 | 1.45869 | 0.02026 | -- | -- |
| Corrected Total | 89 | 3.56452 | -- | -- | -- |
| **Factor A= species** |  |  |  |  |  |
| **Factor B= S and S doses** |  |  |  |  |  |
| At the 0.05 level. the population means of Factor A are significantly different | | | | |  |
| At the 0.05 level. the population means of Factor B are significantly different | | | | |  |
| At the 0.05 level. the interaction between Factor A and Factor B is significant | | | | |  |

**Table 12S. Comparison between species in root Se/S ratio in relation to the S-Se dose applied. Values in bold indicate significant differences for P<0.05.**

|  | ***B. juncea*** | | | | | | | | | |
| --- | --- | --- | --- | --- | --- | --- | --- | --- | --- | --- |
|  |  | *S0Se0* | *S0Se10* | *S0Se20* | *S0.5Se0* | *S0.5Se10* | *S0.5Se20* | *S5Se0* | *S5Se10* | *S5Se20* |
| ***S. pinnata*** | *S0Se0* | 1 | 0.99998 | 1 | 1 | 1 | 1 | 1 | 1 | 1 |
|  | *S0Se10* | 0.99991 | 1 | 1 | 0.99991 | 0.99998 | 1 | 0.99991 | 0.99994 | 1 |
|  | *S0Se20* | 0.99991 | 0.99994 | 0.99996 | **5.30E-08** | **1.03E-06** | **3.54E-07** | **5.30E-08** | **6.64E-08** | **9.93E-08** |
|  | *S0.5Se0* | **5.30E-08** | **5.89E-08** | **6.11E-08** | 1 | 0.9994 | 1 | 1 | 1 | 1 |
|  | *S0.5Se10* | 1 | 1 | 1 | 1 | 1 | 1 | 1 | 1 | 1 |
|  | *S0.5Se20* | 0.99999 | 1 | 1 | 0.99999 | 1 | 1 | 0.99999 | 0.99999 | 1 |
|  | *S5Se0* | 1 | 0.99997 | 1 | 1 | 1 | 1 | 1 | 1 | 1 |
|  | *S5Se10* | 1 | 0.99998 | 1 | 1 | 1 | 1 | 1 | 1 | 1 |
|  | *S5Se20* | 1 | 1 | 1 | 1 | 1 | 1 | 1 | 1 | 1 |
